# Retraction Note: Mesenchymal stem cell-derived exosome miR-542-3p suppresses inflammation and prevents cerebral infarction

**DOI:** 10.1186/s13287-022-03227-x

**Published:** 2023-01-16

**Authors:** Guofeng Cai, Guoliang Cai, Haichun Zhou, Zhe Zhuang, Kai Liu, Siying Pei, Yanan Wang, Hong Wang, Xin Wang, Shengnan Xu, Cheng Cui, Manchao Sun, Sihui Guo, Kunping Jia, Xiuzhen Wang, Dianquan Zhang

**Affiliations:** 1 Hanan Branch of Second Affiliated Hospital of Heilongjiang University of Traditional Chinese Medicine, Harbin, 150001 China; 2 Postdoctoral Research Workstation of Harbin Sport University, Harbin, China; 3 Department of Sport Science and Health, Harbin Sport University, Harbin, 150008 China; 4 Heilongjiang University of Traditional Chinese Medicine, Harbin, 150001 China; 5 Department of Rehabilitation Medicine, Shenzhen Longhua District Central Hospital, Shenzhen, Guangdong Province China

**Retraction Note To: Stem Cell Research & Therapy (2021) 12:2** 10.1186/s13287-020-02030-w

The Editors-in-Chief have retracted this paper after the authors reported discrepancies in the data. Specifically, the authors have stated that the data presented in the paper is incomplete; that the analysis did not use appropriate statistical tests, and that the combination of these factors resulted in inaccurate conclusions that are not supported by the data. Further investigation from the publisher has found that Figure 4B displays the same image as Figure 1D in Gou et al. [1]. The authors were not able to provide a satisfactory explanation for the overlap. All authors agree to this retraction.
